# Phage therapy for multidrug-resistant *Serratia marcescens* infection: mechanisms, preclinical evidence, and translational challenges

**DOI:** 10.3389/fmicb.2026.1959850

**Published:** 2026-10-20

**Authors:** Shengting Zhang, Huili Tao, Sha Zhao, Yunlin Wei

**Affiliations:** 1 School of Ethnic Medicine, Yunnan Minzu University, Kunming, China; 2 Faculty of Life Science and Technology, Kunming University of Science and Technology, Kunming, China

**Keywords:** anti-phage defense systems, clinical feasibility, multidrug resistance, phage therapy, phage–bacteria interactions, *Serratia marcescens*

## Abstract

Multidrug-resistant and carbapenem-resistant *Serratia marcescens* (*S. marcescens*) infections are increasingly difficult to treat because of intrinsic and acquired antimicrobial-resistance mechanisms, limited therapeutic options, and the organism’s capacity to persist within biofilms. Phage therapy has therefore emerged as a potential precision antimicrobial strategy against refractory *S. marcescens* infections. This narrative review critically examines the molecular basis, preclinical development, and translational potential of phage therapy targeting multidrug-resistant *S. marcescens*. Particular emphasis is placed on phage adsorption and receptor recognition, bacterial anti-phage defense systems, capsule- and biofilm-associated resistance, phage-encoded depolymerases, host-range limitations, phage screening and cocktail design, phage–antibiotic interactions, and available preclinical and clinical evidence. Current findings support the biological plausibility of this approach, particularly when strictly lytic phages are matched to the infecting clinical isolate and delivered locally in combination with antibiotics. However, the clinical evidence remains limited primarily to individual compassionate-use reports, and the therapeutic contribution of phages cannot be clearly distinguished from that of concomitant antibiotics, drainage procedures, or supportive care. Additional barriers include the emergence of phage resistance, variable pharmacokinetics, immune-mediated phage clearance, limited standardized susceptibility testing, and manufacturing and regulatory challenges. Future research should prioritize receptor-informed phage selection, representative clinical isolate panels, rationally designed phage cocktails, standardized pharmacokinetic and pharmacodynamic evaluation, and prospective controlled clinical studies. Precision phage therapy, engineered phages, curated phage libraries, and harmonized regulatory pathways may ultimately facilitate the transition of phage therapy from an individualized salvage intervention to a reproducible treatment strategy for multidrug-resistant *S. marcescens* infections.

## 1 Introduction

Infections caused by multidrug-resistant (MDR) and extensively drug-resistant (XDR) *Serratia marcescens* (*S. marcescens*) have emerged as a serious threat to global public health. Although typically non-pathogenic in healthy individuals, this Gram-negative opportunistic pathogen frequently causes severe, life-threatening infections in immunocompromised patients, those with prolonged hospitalization, or individuals undergoing invasive procedures (Cruz et al., 2024; Maragakis et al., 2008). Epidemiological evidence identifies *S. marcescens* as a leading cause of healthcare-associated infections, particularly in intensive care units (ICUs), neonatal intensive care units (NICUs), and among patients with hematologic malignancies or solid organ transplants. It is commonly implicated in ventilator-associated pneumonia, catheter-related bloodstream infections, complicated urinary tract infections, and postoperative wound infections (Daoudi et al., 2018; Morillo et al., 2016; Von Dolinger Brito et al., 1999). Recent genomic surveillance across 35 countries revealed that *S. marcescens* can be classified into 12 distinct phylogenetic lineages, with lineages Sm5, Sm6, and Sm7 showing strong association with human clinical infections, evidence of niche adaptation, and enrichment for high-risk antimicrobial resistance genes—highlighting their potential as emerging epidemic clones (Matteoli et al., 2021).

The antimicrobial resistance profile of *S. marcescens* is especially concerning. *Serratia marcescens* possesses a chromosomally encoded *ampC* gene whose basal expression contributes to intrinsic resistance to several β-lactam antibiotics, including ampicillin, amoxicillin–clavulanate, and early-generation cephalosporins. Although *ampC* expression may increase through regulatory changes, clinically significant AmpC overexpression is uncommon in *S. marcescens* and has been reported in fewer than approximately 5% of isolates or infections. This risk is considerably lower than that observed in organisms such as the *Enterobacter cloacae* complex. Therefore, the presence of chromosomal *ampC* should not be interpreted as evidence of constitutive high-level AmpC production, and antimicrobial selection for *S. marcescens* infections should generally be guided by antimicrobial susceptibility testing (Weber et al., 2020). More alarmingly, this organism has increasingly acquired mobile genetic elements—such as plasmids, transposons, and integrons—that carry genes encoding carbapenemases (e.g., KPC, NDM, VIM) and extended-spectrum β-lactamases (ESBLs) (Zheng et al., 2025; Wang et al., 2025; Radeva et al., 2024). These acquisitions have rendered *S. marcescens* resistant to nearly all β-lactam agents, including last-resort carbapenems. This resistance translates into stark clinical outcomes: a retrospective study of 242 infected patients demonstrated that MDR strains were significantly less susceptible to β-lactams, aminoglycosides, and fluoroquinolones compared to non-MDR isolates, severely narrowing therapeutic choices (Zhu et al., 2025). Although carbapenem resistance remains less prevalent in *S. marcescens* than in other Enterobacterales like *Klebsiella pneumoniae*, sporadic outbreaks involving carbapenemase-producing strains have been documented worldwide, underscoring its potential for rapid clonal spread in high-risk settings (da Rocha et al., 2025). Regionally, the burden is substantial: in a multicenter Brazilian study conducted between 2020 and 2023, *S. marcescens* was the second most common species among carbapenem-resistant *Enterobacterales* isolated from patient respiratory samples—after only *K. pneumoniae*—accounting for 19.57% of all CRE cases, reflecting its growing role in difficult-to-treat pulmonary infections in Latin America (Fochat et al., 2024).

This escalating resistance severely limits therapeutic options. For carbapenem-resistant *S. marcescens* infections, clinicians are often forced to rely on agents such as tigecycline, polymyxins, or ceftazidime–avibactam. However, these drugs are hampered by suboptimal pharmacokinetics (e.g., poor tissue penetration), significant toxicity (notably nephrotoxicity and neurotoxicity), or the rapid emergence of novel resistance mechanisms—such as *mcr*-mediated polymyxin resistance—undermining their reliability (Zhu et al., 2025; Tootla et al., 2021). Retrospective clinical studies consistently report that patients infected with MDR *S. marcescens* bacteremia have a 28-day all-cause mortality rate as high as 50.0%, which is significantly higher than that of patients infected with sensitive strains (Zhu et al., 2025). Compounding this crisis, the pipeline for new systemic antibiotics active against Gram-negative pathogens remains sparse, with few approvals in the past decade. The traditional paradigm of countering resistance with newer antibiotics is no longer sustainable.

Against this backdrop, phage therapy is experiencing renewed scientific and clinical interest. Phage s are viruses that specifically infect and lyse bacteria. They offer several distinctive advantages: exquisite host specificity, the ability to self-amplify at the site of infection, minimal disruption to the host microbiome, and, critically, the capacity to penetrate and disrupt bacterial biofilms (Topka-Bielecka et al., 2021; Tian et al., 2021; Kunisch et al., 2024). Notably, certain *S. marcescens*-targeting phages encode depolymerase enzymes capable of degrading extracellular polymeric substances (EPS), thereby enhancing bacterial clearance within biofilm communities (Kunisch et al., 2024).

Given the urgent unmet need and the promising potential of phage-based interventions, a comprehensive synthesis of current knowledge is warranted. This review aims to critically evaluate the microbiological basis of *S. marcescens* infections, characterize available anti-*Serratia* phages and their mechanisms of action, summarize preclinical and emerging clinical evidence, discuss key translational challenges, and outline future directions. By integrating these facets, we seek to provide an evidence-based foundation for the rational development and clinical application of phage therapy against MDR *S. marcescens*.

Despite the growing number of reviews addressing phage therapy for MDR bacterial infections, most have focused broadly on major ESKAPE pathogens or on the general principles of therapeutic phage development. Consequently, the organism-specific challenges associated with *Serratia marcescens*, including marked receptor diversity, capsule- and biofilm-mediated protection, multiple anti-phage defense systems, and the limited availability of clinically validated phages, have not been comprehensively integrated within a single translational framework.

This review addresses this knowledge gap by specifically examining phage therapy against MDR *S. marcescens*. In contrast to previous broad reviews, it links the molecular basis of phage adsorption and bacterial resistance with phage screening, biofilm-associated infections, phage–antibiotic interactions, preclinical efficacy, emerging clinical experience, manufacturing and regulatory barriers, and future therapeutic development. Accordingly, the review involves the mechanisms of phage-mediated bacterial killing, the evolution of phage resistance, the role of phage-encoded depolymerases in biofilm disruption, strategies for phage–antibiotic synergy, criteria for preclinical selection and safety assessment, available clinical and compassionate-use evidence, and the principal challenges affecting clinical translation. The novelty of this review lies in its organism-specific and translational analysis of phage therapy for MDR and XDR *S. marcescens*. Unlike previous reviews that discuss phage therapy mainly from a broad, pathogen-independent perspective, we integrate the distinctive biological features of *S. marcescens*, including receptor heterogeneity, capsule- and biofilm-mediated protection, phage-defense systems, and the emergence of phage resistance, with practical strategies for phage discovery, characterization, formulation, and therapeutic application. In addition, this review provides a comprehensive framework linking phage–antibiotic interactions, depolymerase-mediated biofilm disruption, preclinical efficacy, compassionate-use and clinical evidence, manufacturing requirements, and regulatory challenges. By bringing these microbiological, therapeutic, and translational aspects together, this work identifies the major barriers to the clinical development of anti-*Serratia* phages and proposes priorities for designing effective and clinically deployable phage-based interventions.

## 2 Literature search strategy

This narrative review was conducted to identify and critically synthesize the available literature concerning the molecular interactions between bacteriophages and *Serratia marcescens*, the preclinical development of phage-based therapeutic strategies, and the current evidence and translational challenges associated with their potential application against multidrug-resistant (MDR) *S. marcescens* infections. A literature search was performed in PubMed/MEDLINE, Web of Science, and Scopus from database inception to March 2026. Reference lists of relevant original articles and reviews were additionally screened to identify potentially eligible studies not retrieved through the electronic database search.

The search strategy combined terms related to the target organism, bacteriophages, mechanisms of phage-host interaction, preclinical evaluation, and therapeutic application. Search terms included combinations of “*Serratia marcescens*,” “Serratia,” “bacteriophage,” “phage,” “phage therapy,” “phage treatment,” “phage receptor,” “host range,” “phage resistance,” “anti-phage defense,” “CRISPR-Cas,” “restriction-modification,” “CBASS,” “abortive infection,” “biofilm,” “depolymerase,” “phage-antibiotic combination,” “preclinical,” “animal model,” “clinical application,” and “human infection.” Boolean operators were adapted according to the requirements of each database. Because direct clinical evidence specifically concerning phage therapy for *S. marcescens* is exceptionally limited, additional targeted searches were conducted using combinations of “*Serratia marcescens*,” “bacteriophage,” “phage therapy,” “phage treatment,” “clinical application,” and “human infection.”

Studies were considered eligible when they provided information directly relevant to at least one of the principal domains addressed in this review: (1) experimentally characterized interactions between bacteriophages and *S. marcescens*, including receptor recognition, phage resistance, bacterial anti-phage defense systems, and phage countermeasures; (2) isolation, characterization, host-range determination, biofilm activity, genomic safety assessment, or other preclinical evaluation of phages active against *S. marcescens*; (3) *in vivo* evaluation of anti-*S. marcescens* phages in animal or invertebrate infection models; or (4) clinical administration of phages for treatment of human *S. marcescens* infection. Original experimental studies, genomic or molecular investigations, preclinical studies, and human case reports or clinical studies were therefore prioritized.

Studies involving other *Serratia* species or other Gram-negative bacterial pathogens were included selectively when they provided mechanistic or translational information for which direct *S. marcescens*-specific evidence was unavailable. Such evidence was clearly distinguished from organism-specific evidence and was used only to inform general principles relating to phage biology, delivery, pharmacokinetics/pharmacodynamics, phage-antibiotic interactions, manufacturing, safety, or clinical implementation. Evidence derived from other bacterial species was not interpreted as direct evidence of therapeutic efficacy against *S. marcescens*.

Studies were excluded when they were unrelated to bacteriophages or *Serratia* biology, focused exclusively on temperate phages without relevance to phage–host mechanisms or therapeutic development, lacked sufficient methodological or experimental information to support the conclusions discussed, or addressed bacterial species without providing a clearly relevant mechanistic or translational contribution. Duplicate records, conference abstracts lacking adequate study details, editorials, commentaries, and non-primary reports were generally excluded from the evidentiary synthesis, although authoritative reviews, regulatory documents, and consensus or guidance publications were consulted when necessary to provide contextual information regarding therapeutic-phage development, quality control, manufacturing, or regulatory considerations.

Because this study was designed as a narrative rather than a systematic review, study selection and evidence synthesis were guided by relevance to the predefined thematic domains rather than by formal meta-analytic eligibility criteria. Particular emphasis was placed on distinguishing direct *S. marcescens*-specific evidence from indirect evidence derived from related organisms. Within the translational assessment, evidence was therefore considered hierarchically as: (i) direct human evidence involving treatment of *S. marcescens* infection; (ii) *S. marcescens*-specific preclinical evidence obtained from *in vitro* or experimental infection models; and (iii) indirect mechanistic or translational evidence from other bacterial pathogens. This approach was used to avoid over-extrapolation from heterogeneous phage–host systems and to ensure that conclusions regarding therapeutic potential remained proportional to the strength and directness of the available evidence.

## 3 Molecular basis of phage–*S. marcescens* interactions

The interaction between bacteriophages and *Serratia marcescens* is strongly influenced by species- and strain-specific variation in lipopolysaccharide structure, capsular polysaccharides, flagellar expression, biofilm formation, and anti-phage defense systems. These features determine whether a phage can adsorb, replicate, disseminate through the bacterial population, and retain activity under clinically relevant conditions. Accordingly, the following sections focus on how the distinctive surface architecture and defense repertoire of *S. marcescens* shape phage host range, resistance development, and therapeutic performance.

### 3.1 Phage receptor recognition in *Serratia marcescens* and its therapeutic implications

The bactericidal activity of virulent phages against *Serratia marcescens* begins with adsorption, a highly selective process in which phage receptor-binding proteins recognize specific structures exposed on the bacterial surface. Because successful adsorption is a prerequisite for genome delivery and productive infection, receptor identity is a major determinant of phage host range and consequently of therapeutic specificity. Importantly, although lipopolysaccharides, outer-membrane proteins, flagella, pili, and capsular polysaccharides can serve as receptors for phages infecting different Gram-negative bacteria, receptor usage cannot be extrapolated from other bacterial species to *S. marcescens*. Experimental evidence in *S. marcescens* specifically supports lipopolysaccharide (LPS), including its O-antigen component, and flagella as phage receptors.

The most direct biochemical evidence concerns bacteriophage κ. Montilla et al. demonstrated that κ adsorbed to outer-membrane fractions prepared from susceptible *S. marcescens* cells. Receptor activity was retained after proteinase K treatment, arguing against a proteinaceous primary receptor, whereas purified LPS was capable of interacting with and inactivating the phage. These experiments identified LPS as the primary receptor for κ in *S. marcescens* (Montilla et al., 1991). Genetic and phenotypic evidence subsequently reinforced the importance of the LPS O-antigen. Palomar et al. isolated spontaneous *S. marcescens* mutants resistant to three LPS-dependent phages—κ, FSB3, and FSB55—and showed that these resistant derivatives were defective in the O-side chain of LPS (Palomar et al., 1993). Thus, alteration or loss of the O-polysaccharide can prevent productive infection by these phages. Further evidence was obtained with phage ϕIF3 in *S. marcescens* Db11. This phage formed plaques on the parental strain but failed to infect the Db11 20C2 mutant, which contains a disruption of an O-antigen transporter and displays an altered LPS phenotype. Adsorption to the LPS-defective mutant was also strongly impaired. Moreover, only two of 25 additional Db11 phages tested retained the ability to infect this mutant, indicating that LPS-dependent recognition is common among phages isolated against this particular *S. marcescens* background (Petty et al., 2006). Collectively, these biochemical, adsorption, and resistant-mutant experiments establish LPS/O-antigen as a bona fide receptor class for several *S. marcescens* phages.

A second experimentally demonstrated receptor is the bacterial flagellum. Bacteriophage χ is a classical flagellotropic phage capable of infecting motile *S. marcescens*, with early studies linking χ susceptibility to the presence and function of flagella (Iino and Mitani, 1967). More recent genetic experiments provide direct confirmation in *S. marcescens* ATCC 274: deletion of *fliC*, which encodes flagellin, generated a flagellin-deficient strain resistant to χ infection. Consequently, the flagellar filament is required for productive χ infection in this host (Esteves and Scharf, 2024). χ initially interacts with the flagellum, and flagellar rotation facilitates movement of the phage toward the bacterial cell surface, after which irreversible adsorption and genome delivery can occur. These findings provide a mechanistically distinct example from LPS-dependent phages and demonstrate that different *S. marcescens* phages can exploit structurally and functionally unrelated surface receptors.

The available evidence therefore does not support treating all commonly reported Gram-negative phage receptors as established receptors in *S. marcescens*. In particular, although porins such as OmpF/OmpC and bacterial pili are well-characterized phage receptors in other species, direct experimental evidence identifying them as receptors for *S. marcescens* phages is presently insufficient. Similarly, the receptor has not been experimentally characterized for many recently isolated *S. marcescens* phages. Distinguishing experimentally validated receptors from predicted or uncharacterized receptor interactions is particularly important when evaluating phages for therapeutic use.

Receptor specificity has direct consequences for therapeutic host range. The LPS structure of *S. marcescens* is heterogeneous among strains, particularly within the O-antigen, and modification or loss of the relevant polysaccharide may reduce or abolish adsorption of an LPS-dependent phage. Likewise, alterations in flagellar production, flagellin structure, or motility may confer resistance to flagellotropic phages such as χ. Surface-associated structures can also be affected by growth conditions or masked by extracellular material, further modifying receptor accessibility. Consequently, a phage that efficiently infects one clinical *S. marcescens* isolate may show weak or absent activity against another isolate of the same species. Taxonomic identification alone is therefore insufficient for therapeutic phage selection; isolate-specific susceptibility and adsorption testing remain essential. Where receptor information is available, cocktails containing phages that use mechanistically distinct receptors may additionally reduce the likelihood that modification or loss of a single surface structure will generate cross-resistance to all members of the formulation.

Release of progeny particles is typically mediated by a coordinated phage-encoded lysis system. In many dsDNA phages, holins accumulate in the bacterial inner membrane and, at a genetically determined time, permeabilize the membrane, enabling phage endolysins to reach and hydrolyze the peptidoglycan layer (Baldvinsson et al., 2014; Xu et al., 2025). In Gram-negative bacteria such as *S. marcescens*, disruption of the outer membrane must also accompany degradation of the peptidoglycan for efficient cell lysis, and many phages employ additional lysis proteins, including spanins, to complete this process. Loss of envelope integrity results in bacterial rupture and release of newly assembled virions, which can subsequently infect neighboring susceptible cells. This sequence of receptor recognition, genome delivery, intracellular replication, assembly, and host-cell lysis constitutes the basis of the antibacterial activity illustrated in Figure 1.

**Figure 1:**
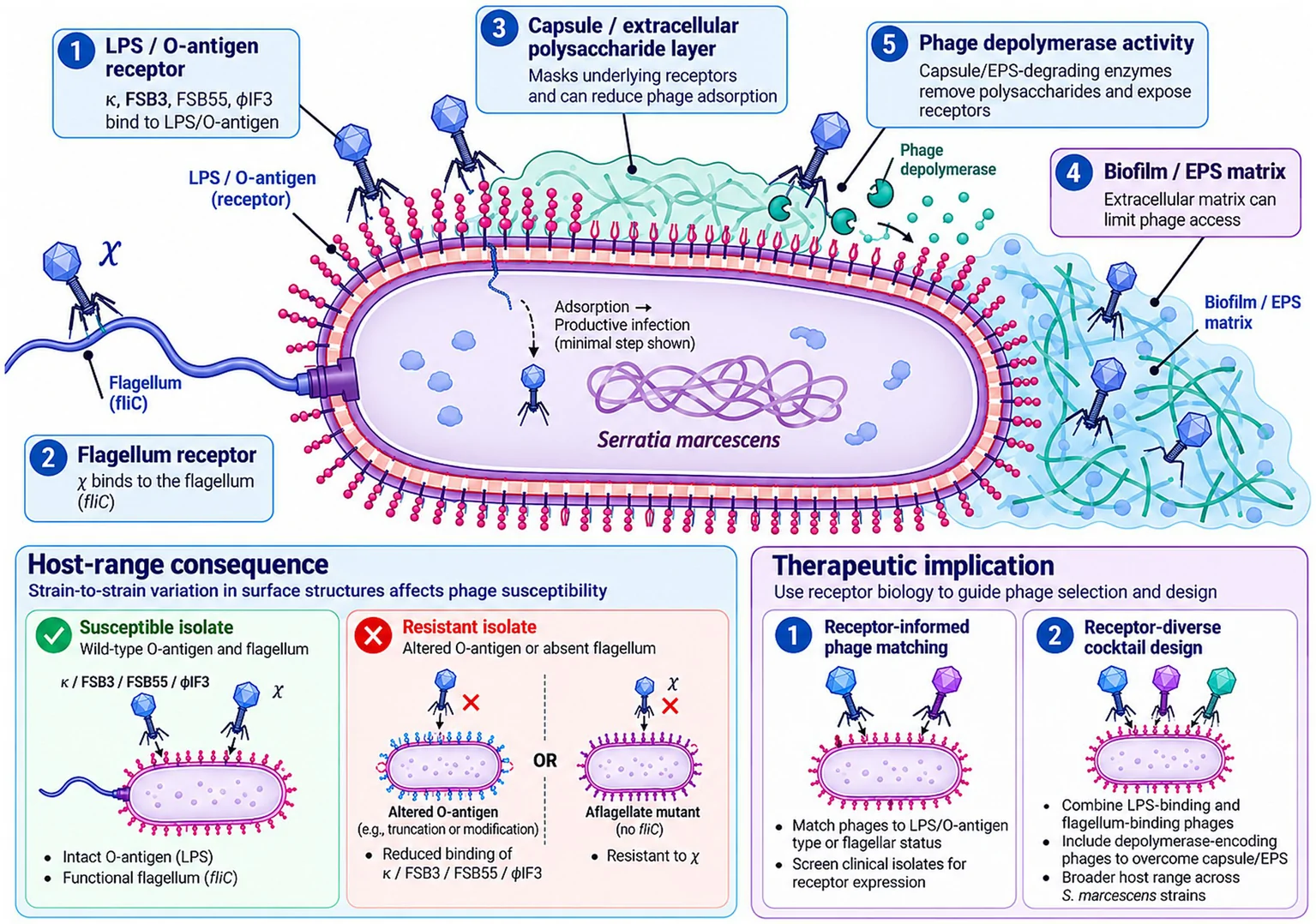
*Serratia marcescens*-specific determinants of productive phage infection and their therapeutic implications. Schematic representation of surface-associated factors influencing bacteriophage susceptibility in *S. marcescens*. Experimentally supported receptor interactions include recognition of lipopolysaccharide (LPS)/O-antigen by phages κ, FSB3, FSB55, and ϕIF3, and flagellum-dependent infection by phage χ. Capsule and extracellular polymeric substance (EPS) layers may reduce receptor accessibility and limit phage adsorption, whereas phage-associated depolymerases can facilitate degradation of capsular or extracellular polysaccharides and thereby improve access to underlying receptors. Strain-to-strain variation in O-antigen structure and flagellar expression may consequently generate marked differences in phage susceptibility and host range. These features provide a rationale for isolate-specific phage matching and for combining phages with complementary receptor specificities in therapeutic cocktails. *fliC*, flagellin structural gene; EPS, extracellular polymeric substances; LPS, lipopolysaccharide.

From a therapeutic perspective, productive lytic infection provides phages with a distinctive pharmacodynamic property. For therapeutic application against *S. marcescens*, the principal implication of these findings is that successful infection depends first on compatibility between the phage and the surface phenotype of the infecting isolate. Experimental studies demonstrate that modification or loss of LPS/O-antigen can prevent infection by κ, FSB3, FSB55, and ϕIF3, whereas loss of functional flagella confers resistance to χ. These observations establish receptor availability as a major determinant of phage susceptibility in *S. marcescens* and provide a mechanistic basis for the marked strain-to-strain variation observed in phage host range. Consequently, therapeutic selection should prioritize isolate-specific adsorption and productive-infection assays and, where receptor information is available, combine phages recognizing distinct surface structures to reduce receptor-mediated cross-resistance. Downstream processes such as genome replication, virion assembly, and cell lysis are essential components of productive phage infection but are not discussed here in detail because their molecular mechanisms have not been characterized sufficiently across therapeutic *S. marcescens* phages to support species-specific generalization.

### 3.2 Bacterial anti-phage defense systems

Bacteria have evolved multiple defense mechanisms that interfere with phage infection at successive stages of the viral life cycle, including adsorption, genome entry, replication, transcription, and progeny production. In *Serratia marcescens*, available experimental and genomic evidence indicates that resistance can arise through modification of phage receptors, restriction–modification (R–M) systems, CRISPR–Cas immunity, cyclic-oligonucleotide-based signaling, prophage-associated exclusion, and abortive-infection-like mechanisms. Importantly, however, the distribution of these systems is strain dependent; therefore, the presence or activity of a particular defense mechanism should not be generalized to all *S. marcescens* isolates.

One of the best experimentally documented mechanisms is alteration of cell-surface structures required for phage adsorption. In *Serratia marcescens*, alteration of cell-surface structures involved in phage adsorption represents a well-supported mechanism of phage resistance. LPS has been experimentally identified as the primary receptor for bacteriophage κ (Montilla et al., 1991), while spontaneous mutants selected for resistance to κ, FSB3, and FSB55 exhibited defects in the LPS O-side chain (Palomar et al., 1993). In addition, adsorption and productive infection by phage ϕIF3 were markedly impaired in an *S. marcescens* Db11 mutant with an altered LPS/O-antigen phenotype (Petty et al., 2006). Collectively, these findings demonstrate that modification or loss of LPS/O-antigen can reduce or abolish susceptibility to specific *S. marcescens* phages (Topka-Bielecka et al., 2020). Thus, mutations affecting LPS biosynthesis or assembly may prevent productive adsorption before phage DNA reaches the cytoplasm. This mechanism also illustrates an important evolutionary trade-off: modifications that provide phage resistance can simultaneously alter other envelope-associated phenotypes, including membrane permeability and susceptibility to antimicrobial agents (Topka-Bielecka et al., 2020). Although capsular polysaccharides, extracellular polymeric substances, and biofilms can physically influence phage access to bacterial surfaces, their roles as specific anti-phage mechanisms in *S. marcescens* remain less directly established and should therefore be distinguished from experimentally demonstrated receptor loss or modification.

Once phage DNA has entered the cell, restriction–modification systems provide another potential barrier. A type II R–M system from *S. marcescens*, SmaI, has been molecularly characterized: the genes encoding the cognate restriction endonuclease and DNA-modification methyltransferase were identified by cloning, sequencing, deletion analysis, and insertion mutagenesis (Sritha and Bhat, 2018). The underlying principle is discrimination between appropriately modified host DNA and susceptible foreign DNA, although the effectiveness of individual systems against particular phages depends on both bacterial and phage genotypes. More recent comparative genomics further indicates substantial R–M diversity within *S. marcescens*. Analysis of population structure identified distinct R–M repertoires among major genetic clusters, including enrichment of type IIG and type IV systems in particular lineages, demonstrating that restriction barriers are not uniformly distributed throughout the species (Palmer et al., 2021). Such diversity may contribute to isolate-specific differences in susceptibility to therapeutic phages and provides a potential explanation for why a phage can replicate efficiently in one *S. marcescens* strain but poorly in another.

CRISPR–Cas systems constitute a sequence-specific adaptive defense, but their occurrence in *S. marcescens* is likewise strain dependent. Genomic analysis of four *S. marcescens* strains identified a type I-F CRISPR–Cas system in strain S5 and a type I-E system in strain S8, whereas strains S1 and S13 lacked detectable CRISPR–Cas systems (Samir, 2024). Examination of additional complete *S. marcescens* genomes similarly demonstrated that CRISPR–Cas is present only in a subset of strains. Importantly, several CRISPR spacers showed similarity to mobile genetic elements, and six spacers in the S8 arrays matched sequences from Serratia phage Eta, providing evidence of historical interactions between these bacteria and phages (Samir, 2024). Spacer–phage sequence correspondence alone does not establish the current antiviral activity of a CRISPR system, but it supports its potential involvement in phage recognition.

Functional evidence from the closely studied *Serratia* sp. ATCC 39006 model further demonstrates how CRISPR immunity can determine the outcome of phage infection. This strain carries three native CRISPR–Cas systems—types I-E, I-F, and III-A. During infection by jumbo phage PCH45, the phage genome becomes enclosed within a nucleus-like protein shell that physically excludes the DNA-targeting type I-E and I-F CRISPR complexes. In contrast, the type III-A system remains effective because it recognizes phage RNA in the cytoplasm; disruption experiments demonstrated that Cas7, Cas10, and an accessory nuclease are required for this antiviral activity (Harvey et al., 2018). Although these mechanistic experiments were performed in *Serratia* sp. ATCC 39006 rather than *S. marcescens*, they provide direct evidence within the genus that the effectiveness of CRISPR immunity depends not only on the presence of a CRISPR locus but also on the molecular biology and counter-defense strategy of the infecting phage.

A particularly important recent finding is the characterization of a cyclic-oligonucleotide-based anti-phage signaling system (CBASS) derived from *S. marcescens*. CBASS systems detect phage-associated perturbations and activate intracellular signaling pathways that can terminate infection before productive phage propagation. Biochemical, genetic, structural, and mass-spectrometric analyses of the *S. marcescens* E2-CBASS system demonstrated an unusual regulatory mechanism in which an E2-like enzyme processes and covalently modifies the cGAS-like cyclic-dinucleotide synthase. This sequential “cGASylation” promotes synthase activation and production of the cyclic antiviral signal, ultimately activating a cell-death-associated defensive response (Palomar et al., 1995). These experiments provide molecular evidence that *S. marcescens* can encode sophisticated signaling-based innate immunity in addition to classical R–M and CRISPR systems.

Abortive infection represents another strategy in which phage propagation is blocked at the expense of the infected bacterial cell. Evidence for abortive infection involving *S. marcescens* dates to experiments showing abortive infection following exposure to coliphage P1 (Aucken et al., 1997), although the molecular mechanism underlying that early observation was not defined. More detailed mechanistic information has subsequently emerged from *Serratia* sp. ATCC 39006. Functional genomic analysis identified 32 predicted toxin–antitoxin systems, of which 11 showed evidence of functionality; three belonged to the AbiE abortive-infection/toxin–antitoxin family (Rusinov et al., 2018). Expression of the AbiEii toxin altered cellular tRNA abundance and inhibited bacterial growth, supporting a mechanism in which disruption of translation produces bacteriostasis capable of restricting viral propagation (Rusinov et al., 2018). These observations illustrate how infection-triggered growth arrest or cell death can protect the surrounding bacterial population by preventing completion of the phage reproductive cycle.

Genomic evidence further indicates that anti-phage determinants in *S. marcescens* can occur within horizontally acquired genomic islands. The genome of *S. marcescens* UENF-22GI contains an island encoding several predicted defense-related components, including an ADP-ribosyltransferase-related protein with similarity to abortive-infection proteins, an AbiE-related ATPase, and proteins predicted to participate in DNA modification (Marraffini, 2015). Because these assignments are primarily bioinformatic, they should be regarded as candidate defense systems rather than experimentally validated anti-phage mechanisms in this strain. Nevertheless, the genomic organization is consistent with the broader “defense island” concept, in which multiple anti-phage genes accumulate within genomic hotspots. Recent experimental investigation across the genus *Serratia* identified three such multi-functional defense islands and demonstrated several distinct anti-phage activities, including a TIR-domain-containing system and systems incorporating predicted type VI secretion-associated proteins; some of these mechanisms reduced phage adsorption, whereas others acted after infection (Nussenzweig and Marraffini, 2020). These findings show that the defensive repertoire of *Serratia* is considerably broader than the classical R–M/CRISPR framework.

Collectively, the available evidence portrays anti-phage immunity in *S. marcescens* as a heterogeneous and multilayered phenotype rather than a single conserved pathway. Surface-receptor alteration can block adsorption, R–M and CRISPR systems can discriminate invading nucleic acids, CBASS can initiate infection-responsive antiviral signaling, and abortive-infection-associated systems can terminate cellular activity before completion of the phage life cycle. Moreover, phages themselves can evolve counter-defense mechanisms, meaning that the mere genomic presence of a bacterial defense locus does not necessarily predict resistance to a particular therapeutic phage. Consequently, therapeutic phage selection against *S. marcescens* should combine isolate-specific host-range and efficiency-of-plating assays with adsorption measurements and, where feasible, genomic analysis of bacterial defense systems. Phage cocktails containing viruses that use different receptors and differ in their sensitivity to intracellular defense systems may reduce the probability that a single resistance mechanism will abolish therapeutic activity. These complementary bacterial defense mechanisms and their points of interference with the phage infection cycle are summarized in Figure 2.

**Figure 2:**
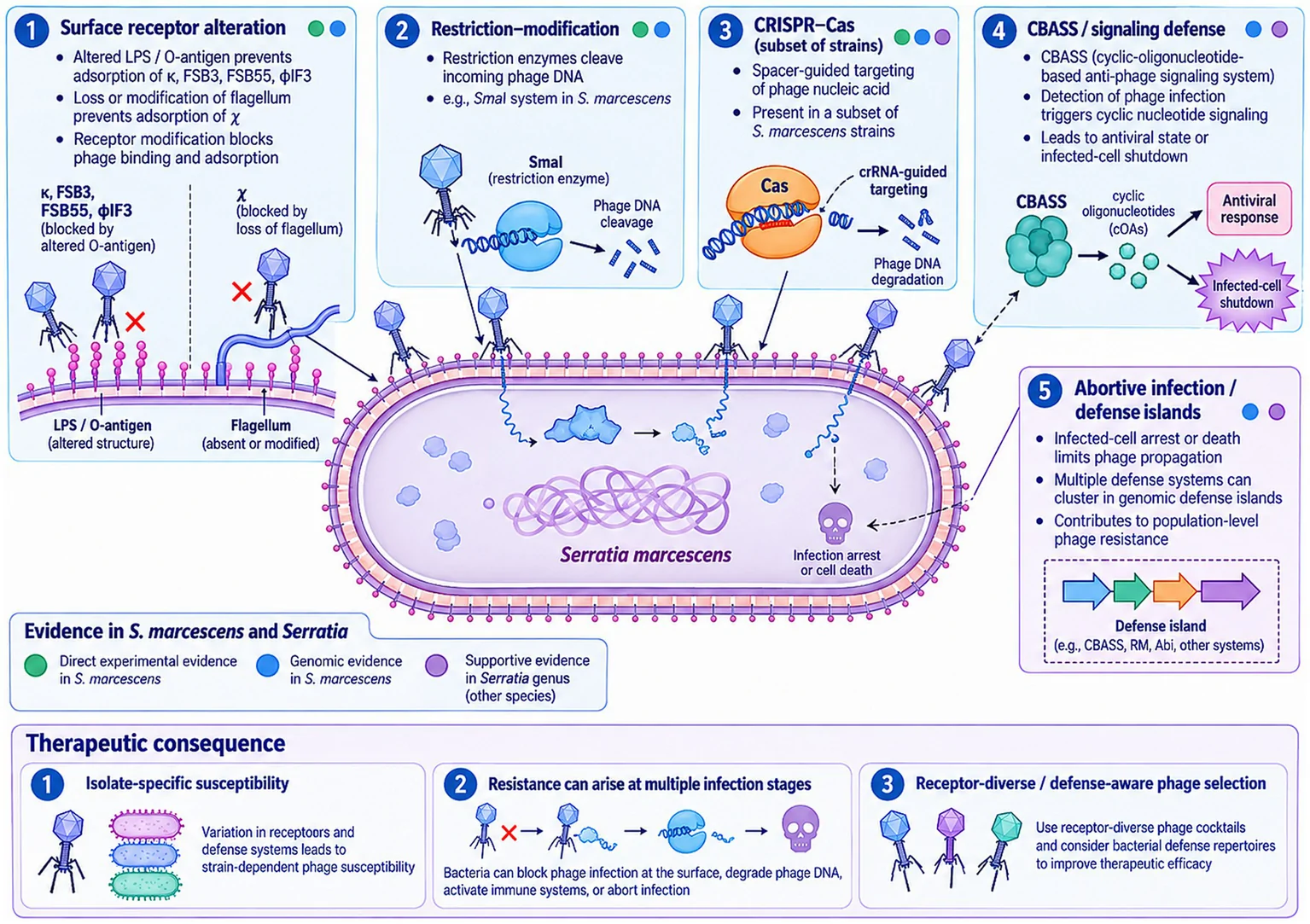
Anti-phage defense mechanisms relevant to *Serratia marcescens* and their implications for therapeutic phage susceptibility. Schematic overview of extracellular and intracellular mechanisms that can restrict phage infection in *S. marcescens*, presented according to the level of organism-specific evidence. Surface receptor alteration, including modification of LPS/O-antigen or loss of functional flagella, can prevent adsorption of receptor-dependent phages. Intracellular defenses include restriction–modification systems, illustrated by the SmaI system; CRISPR–Cas-mediated sequence-specific targeting in subsets of *S. marcescens* strains; cyclic-oligonucleotide-based anti-phage signaling systems such as CBASS; and abortive-infection mechanisms that restrict phage propagation through infection arrest or death of the infected cell. Multiple defense determinants may occur within genomic defense islands. Colored evidence markers distinguish direct experimental evidence in *S. marcescens*, genomic evidence in *S. marcescens*, and supportive evidence derived from other members of the genus *Serratia*. Collectively, receptor and intracellular defense diversity can contribute to isolate-specific susceptibility and supports receptor-diverse and defense-aware approaches to phage selection. CBASS, cyclic-oligonucleotide-based anti-phage signaling system; CRISPR–Cas, clustered regularly interspaced short palindromic repeats and CRISPR-associated proteins; LPS, lipopolysaccharide; RM, restriction–modification.

### 3.3 *Serratia marcescens*-specific phage recognition and countermeasures

The molecular dialogue between phages and *S. marcescens* is uniquely shaped by the pathogen’s prominent surface structures, primarily its lipopolysaccharide (LPS) O-antigen and its thick capsule. These features are not only critical virulence factors but also serve as the primary determinants of phage host specificity and the main targets for phage-encoded countermeasures.

LPS/O-antigen structure varies among *S. marcescens* strains and can critically influence susceptibility to particular bacteriophages. Direct experimental evidence identifies LPS as the receptor for phage κ (Montilla et al., 1991), while O-antigen-defective mutants exhibit resistance to κ, FSB3, and FSB55 (Palomar et al., 1993). Similarly, impaired adsorption of ϕIF3 to an LPS-defective *S. marcescens* Db11 mutant supports an LPS-dependent recognition mechanism for this phage (Petty et al., 2006). Thus, LPS/O-antigen should be regarded as an experimentally supported receptor determinant for several characterized *S. marcescens* phages, rather than as a universally established receptor for *Serratia*-specific phages. Structural variation or loss of these surface polysaccharides may consequently alter adsorption and contribute to strain-dependent phage susceptibility. This tight receptor–ligand interaction forms the basis for precise strain targeting but necessitates careful phage matching in a clinical context.

Beyond the O-antigen, the production of a robust capsular polysaccharide layer is another key defense employed by *S. marcescens* to evade both host immunity and phage predation. This capsule creates a formidable physical barrier that can completely occlude underlying phage receptors, such as LPS or porins, effectively blocking the initial adsorption step of the phage lytic cycle (An et al., 2025). To overcome this barrier, a significant number of characterized *S. marcescens* phages encode specialized virion-associated enzymes known as depolymerases. These enzymes are often located at the distal end of the phage tail fibers or baseplate and are activated upon contact with the bacterial surface (Aframian and Eldar, 2023).

Depolymerases exhibit high substrate specificity and can degrade glycosidic linkages within capsular polysaccharides, *O*-antigen structures, or other components of the extracellular polymeric matrix (Lee et al., 2023). This enzymatic activity can locally disrupt the protective capsule or extracellular matrix, thereby exposing otherwise inaccessible bacterial surface receptors and facilitating phage adsorption and genome delivery. In addition, degradation of capsule or extracellular polymeric substances can reduce the physical barrier and local matrix viscosity surrounding bacterial cells, thereby facilitating the passive diffusion and access of phage particles to susceptible bacterial receptors (Nehme et al., 2007; Li et al., 2021). By degrading capsular or extracellular polymeric components, phage-associated depolymerases may also facilitate penetration into biofilm-associated bacterial populations and improve access to cells embedded in the biofilm matrix. This property may be particularly relevant to *S. marcescens* infections involving biofilm formation, including device-associated infections. Accordingly, the presence and activity of a functional depolymerase may represent a potentially advantageous characteristic when selecting phages against encapsulated or biofilm-forming *S. marcescens* strains; however, its presence alone cannot be considered as a reliable predictor of therapeutic efficacy, which is additionally influenced by host range, adsorption efficiency, bacterial susceptibility, phage replication kinetics, biofilm architecture, and *in vivo* conditions. This molecular interplay defines the specificity and evolutionary constraints of *Serratia*–phage interactions, shaping both bacterial resistance and phage counter-adaptation in natural environments.

## 4 Preclinical development: screening optimization and safety assessment of phages

Preclinical development of phage therapy against *Serratia marcescens* remains less advanced than that reported for several other MDR Gram-negative pathogens. The available literature consists mainly of individual phage-isolation and characterization studies, with relatively few investigations evaluating activity across representative clinical isolate panels, mature biofilms, animal infection models, or clinically relevant phage–antibiotic combinations. Therefore, the preclinical evidence should be interpreted not only according to general phage-selection principles but also in relation to the specific biological characteristics of *S. marcescens*, including *O*-antigen and capsular diversity, strong biofilm formation, and the presence of multiple anti-phage defense systems.

### 4.1 Phage isolation, characterization, and safety assessment

The development of clinically viable phage therapeutics begins with the isolation of virulent, host-specific phages capable of efficiently lysing target *S. marcescens* strains. Candidate phages are typically sourced from environmental reservoirs—such as hospital wastewater, sewage, soil, or clinical specimens—where phage–bacteria coevolution is intense (Anderson et al., 2025; Hughes et al., 1998). Based on the available *S. marcescens* phage literature and general principles of therapeutic-phage development, a proposed practical stepwise framework can be used to organize preclinical candidate selection and evaluation. This framework comprises: (1) enrichment and isolation of candidate phages using relevant bacterial hosts; (2) preliminary phenotypic characterization, including plaque morphology and lytic activity; (3) genomic characterization and safety screening for undesirable genetic determinants, including lysogeny-associated, virulence, and antimicrobial-resistance genes; (4) host-range and lytic-activity assessment using a representative and genetically diverse panel of contemporary clinical *S. marcescens* isolates; and (5) subsequent evaluation of promising candidates in appropriate *in vivo* infection models. This framework is intended as a practical organizational approach rather than as an established or universally standardized screening protocol (Figure 3). No internationally harmonized guideline establishes a universal numerical threshold for the number of clinical isolates required for bacteriophage host-range characterization. Rather than relying on an arbitrary minimum number of strains, the composition of the test panel should be scientifically justified according to the intended therapeutic application and should adequately represent the epidemiological and genetic diversity of the target bacterial population. Accordingly, candidate anti-*S. marcescens* phages should be evaluated against a representative and genetically diverse panel of contemporary clinical isolates that, where feasible, encompasses distinct clonal lineages, antimicrobial-resistance profiles, surface-antigen or serotype variants, infection sources, and geographic and temporal origins. The number of isolates required will depend on the purpose and stage of phage development, with progressively larger and more systematically structured collections needed to support claims of broad host-range coverage or advancement toward clinical evaluation. Thus, panel representativeness and diversity, rather than a fixed numerical cut-off, should guide host-range assessment.

**Figure 3:**
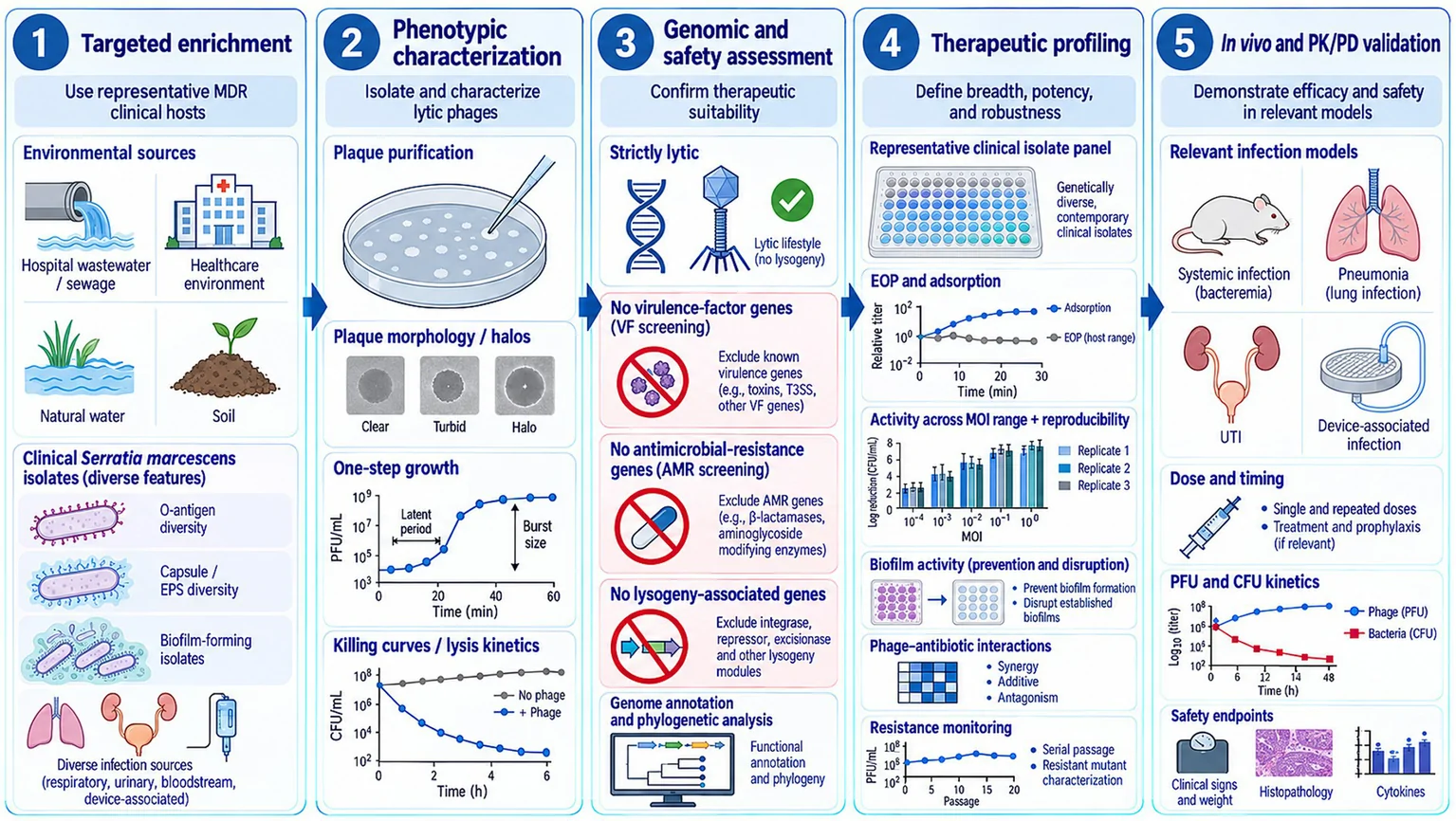
Proposed practical stepwise framework for the preclinical selection and evaluation of therapeutic bacteriophages against *Serratia marcescens*. Candidate phages are enriched using clinically relevant host isolates, followed by phenotypic characterization, genomic and safety assessment, therapeutic profiling against a representative and genetically diverse clinical isolate panel, and subsequent validation in relevant *in vivo* infection models. Therapeutic profiling includes quantitative host-range assessment, adsorption and EOP testing, evaluation across different MOIs, biofilm activity, phage–antibiotic interactions, reproducibility, and resistance monitoring. *In vivo* studies should assess dose and timing, phage and bacterial kinetics, and relevant safety endpoints. AMR, antimicrobial resistance; CFU, colony-forming units; EOP, efficiency of plating; MOI, multiplicity of infection; MDR, multidrug-resistant; PFU, plaque-forming units; PK/PD, pharmacokinetics/pharmacodynamics.

Initial enrichment is performed by co-culturing environmental samples with the target bacterial strain, followed by plaque purification using the double-agar overlay method to obtain clonal isolates (Noreika et al., 2023). Purified phages undergo preliminary phenotypic characterization, including determination of the one-step growth curve to define latent period and burst size, and host-range assessment against the selected clinical-isolate panel (Marongiu et al., 2025). Because clearing in a spot assay does not necessarily demonstrate productive phage infection, spot testing is best regarded as an initial screening method; putatively susceptible isolates should subsequently be confirmed by plaque formation and quantitative efficiency-of-plating (EOP) analysis relative to a defined reference host.

Genomic integrity and biosafety constitute the decisive gatekeeping criteria. All candidate phages must undergo complete genome sequencing, followed by comprehensive bioinformatic screening against curated databases of virulence factors, antibiotic resistance genes, and mobile genetic elements (Ge et al., 2026). Critically, the genome must lack any homology to known toxin, superantigen, or immune evasion genes that could be horizontally transferred to the host bacterium. Equally essential is the unequivocal confirmation of a strictly lytic lifecycle. Annotation must demonstrate the absence of hallmark temperate phage genes—including integrase (*int*), repressor (*cI*), excisionase (*xis*), and other lysogeny maintenance modules (Azizian et al., 2015; Wu et al., 2025). The presence of such elements raises unacceptable risks of lysogenic conversion, unintended gene transfer, or spontaneous induction under stress conditions. Only phages classified as obligately virulent based on both genomic architecture and functional assays (e.g., inability to form stable lysogens) are considered suitable for therapeutic development.

Finally, phylogenetic placement and comparative analysis of structural, replication, and lysis genes can provide additional information regarding phage identity, biological characteristics, and evolutionary relationships. Collectively, the integration of phenotypic characterization, host-range assessment, genomic analysis, and appropriate preclinical evaluation provides a rational framework for selecting therapeutic phage candidates and for generating the safety and characterization data required for subsequent translational development.

### 4.2 Strategic screening for therapeutically relevant phages against *S. marcescens*

The preclinical selection of anti-*S. marcescens* phages must prioritize candidates capable of overcoming the pathogen’s primary physical defenses: a thick capsular layer and robust biofilm formation. Two strategic refinements, supported by recent experimental evidence, significantly enhance the likelihood of identifying therapeutically viable candidates.

To further broaden therapeutic coverage, phage isolation should not rely on a single enrichment host. Instead, multiple genetically and phenotypically diverse clinical *S. marcescens* isolates should be incorporated into parallel or sequential enrichment procedures. These panels should represent different antimicrobial-resistance profiles, phylogenetic lineages, *O*-antigen structures, capsule phenotypes, biofilm-forming capacities, and infection sources. Sequential propagation across several clinical isolates may enrich phages capable of infecting a broader range of clinically relevant strains and reduce the selection bias introduced by using only one laboratory or patient-derived isolate. Candidate phages should subsequently be evaluated against an expanded clinical isolate collection using quantitative efficiency-of-plating assays, liquid-culture killing curves, and time-kill analyses rather than spot testing alone. Host-range matrices generated from these assays can identify complementary phages and distinguish productive infection from nonspecific lysis from without. Priority should be given to phages that retain high lytic efficiency across epidemiologically distinct isolates and remain active under physiologically relevant conditions, including in biofilms and in the presence of serum or clinically relevant antibiotics. Multiplicity of infection (MOI) should also be explicitly considered during quantitative characterization of candidate phages. *In vitro*, the input MOI is commonly expressed as the ratio of plaque-forming units (PFU) added to viable bacterial cells (CFU) at the start of the assay; however, this value should not be interpreted as the actual number of phages infecting each bacterial cell. The effective multiplicity is influenced by adsorption kinetics, receptor accessibility, bacterial density and physiological state, exposure time, and environmental conditions. Consequently, phage activity should be evaluated across multiple input MOIs rather than at a single arbitrarily selected ratio, and MOI should always be reported together with the absolute phage concentration, bacterial inoculum, treatment volume, and timing of phage addition. Comparison across different MOIs can distinguish candidates that suppress bacterial growth through efficient replication at relatively low phage input from those requiring disproportionately high phage concentrations. Moreover, rapid bacterial clearing at very high phage concentrations should be interpreted cautiously because lysis from without may occur without productive intracellular replication; therefore, reductions in optical density should, where appropriate, be corroborated by plaque formation, viable-count measurements, and demonstration of progeny phage production. Reproducibility represents an equally important criterion for therapeutic phage selection. Phage susceptibility and killing assays should therefore be performed using technical replicates within individual experiments and independent biological replicates conducted on different occasions using independently prepared bacterial cultures and, where feasible, independent phage preparations. Experimental variables that strongly influence phage–host interactions, including bacterial growth phase and starting CFU, phage titer and MOI, culture medium, divalent-ion supplementation, incubation temperature, adsorption period, assay duration, and the bacterial reference strain, should be standardized and reported in sufficient detail to permit replication. Reproducibility should be assessed not only by the presence or absence of bacterial lysis but also by the consistency of quantitative endpoints such as EOP, reduction in viable bacterial counts, suppression of growth curves, latent period, burst size, and emergence of bacterial regrowth. For advanced therapeutic candidates, concordance across different experimental days, operators, phage-production batches, and, where possible, independent laboratories provides substantially stronger evidence of robustness than a single successful *in vitro* experiment. When no individual phage provides adequate strain coverage, rationally designed phage cocktails offer an alternative strategy. Cocktail components should preferably recognize different bacterial receptors, exhibit complementary host ranges, and retain activity against mutants resistant to the other constituent phages. Such receptor-diverse combinations may increase coverage of heterogeneous clinical isolates and reduce the likelihood that alteration of a single receptor will produce resistance to the entire formulation. However, cocktail development should include testing for phage–phage interference, stability during storage and administration, relative replication kinetics, and the emergence of cross-resistant bacterial populations. Periodic reassessment against contemporary clinical isolates may also be required to ensure that cocktail composition remains aligned with changes in circulating *S. marcescens* lineages.

First, the choice of host strain during enrichment is paramount. Direct use of clinical, MDR *S. marcescens* isolates rather than standard laboratory strains ensures that recovered phages are pre-adapted to infect and lyse the most challenging clinical variants (Weber et al., 2020; Usman and Christina, 2024). This principle was clearly demonstrated in the isolation of phage WSPA. Researchers specifically employed an MDR clinical isolate of *S. marcescens* as the enrichment host, which enabled the successful recovery of a novel phage exhibiting potent lytic activity against a broad panel of contemporary MDR strains. Had a non-clinical or non-encapsulated strain been used, such a candidate could have been overlooked entirely. Studies of phages active against *S. marcescens* indicate that the choice of enrichment host strongly influences the clinical relevance of the recovered candidates. The reported activity of WSPA against contemporary MDR isolates supports the use of patient-derived strains during phage isolation rather than reliance on laboratory reference strains alone. However, activity against a limited isolate panel does not necessarily predict population-level coverage because clinical *S. marcescens* strains may differ substantially in surface receptors, capsular composition, biofilm phenotype, and intracellular anti-phage defenses. Accordingly, candidate phages should be tested against genetically and geographically diverse clinical collections, including isolates from respiratory, bloodstream, urinary, wound, and device-associated infections.

Second, plaque morphology provides a rapid functional indicator of depolymerase activity. Phages that produce a translucent halo surrounding the central lysis zone typically encode virion-associated enzymes capable of degrading capsular polysaccharides or EPS. This enzymatic capability directly enhances biofilm penetration and disruption. A compelling example is the podovirus UFV01, isolated using a clinical *S. marcescens* strain. Although strictly specific to *S. marcescens*, UFV01’s tail fiber-associated depolymerase degraded EPS components shared across Enterobacteriaceae, leading to significant reduction of preformed *Escherichia coli* biofilms without infecting the cells (Elsayed et al., 2023). This bystander effect, mediated solely by matrix degradation, underscores why halo formation is a more reliable predictor of therapeutic potential than conventional metrics such as plaque size or burst kinetics. By anchoring early screening on clinically relevant host strains and depolymerase-linked phenotypes, this focused approach efficiently enriches for phages with genuine activity against resilient, biofilm-associated *S. marcescens* infections, thereby providing a robust foundation for subsequent genomic safety evaluation and *in vivo* testing.

## 5 Current clinical evidence, translational gaps, and research priorities

Direct clinical evidence for phage therapy against *S. marcescens* remains extremely limited. As identified through the literature search described in Section 2, only a single published human therapeutic application specifically targeting *S. marcescens* was identified: a 2025 case report describing treatment with phage Spe5P4 in a 59-year-old patient with refractory multidrug-resistant *S. marcescens* pulmonary infection. No prospective clinical trials, controlled studies, or additional published human cases specifically evaluating phage therapy for *S. marcescens* were identified. Accordingly, the available human evidence remains restricted to an individual clinical application and should be interpreted as exploratory evidence of therapeutic administration and preliminary safety rather than evidence establishing clinical efficacy or generalizable clinical feasibility.

Given the scarcity of human data, the present section critically evaluates the available evidence according to three predefined levels: (i) direct clinical evidence obtained from patients treated for *S. marcescens* infection; (ii) *S. marcescens*-specific preclinical evidence obtained from experimental infection models; and (iii) indirect translational evidence derived from phage-therapy studies involving other Gram-negative pathogens. Evidence in the third category is used solely to inform general principles of phage delivery, safety, pharmacokinetics/pharmacodynamics, phage–antibiotic combination therapy, and clinical implementation and is not considered direct evidence of efficacy against *S. marcescens*.

- Direct clinical evidence in *S. marcescens*. Direct human evidence remains limited to the 2025 patient-level report describing treatment of a refractory MDR *S. marcescens* pulmonary infection with the lytic phage Spe5P4. In that case, local intrapleural and nebulized phage administration was associated with microbiological and clinical improvement without reported phage-related toxicity. However, Spe5P4 was administered concomitantly with meropenem and amikacin, together with drainage and supportive care; therefore, the independent contribution of phage therapy cannot be determined. This report provides an exploratory demonstration that individualized phage-antibiotic therapy could be administered in a patient with refractory MDR *S. marcescens* infection without reported phage-related toxicity. However, because the intervention was uncontrolled and incorporated concomitant antibiotics, drainage, and supportive care, it does not establish the independent efficacy, safety profile, or generalizable clinical feasibility of phage therapy against *S. marcescens*. No controlled clinical trial or case series specifically establishing therapeutic efficacy against this pathogen has yet been identified.
- *S. marcescens*-specific preclinical evidence. Experimental infection models provide additional organism-specific proof of concept. In a BALB/c mouse model of lethal *S. marcescens* infection, immediate administration of phage φSM9-3Y produced 100% protection, whereas delaying treatment by 60 min reduced protection to approximately 60% despite administration of a higher phage dose, demonstrating that therapeutic outcome is strongly influenced by treatment timing and phage exposure. More recently, the lytic phage Sm10b_1 was evaluated against the clinical bloodstream isolate *S. marcescens* UMH9 in a *Galleria mellonella* infection model. Untreated larvae showed 0% survival, whereas a single dose of 1 × 10^7^ PFU Sm10b_1 administered after infection increased six-day survival to 67% (*p* < 0.0001). Together, these studies demonstrate reproducible *in vivo* antibacterial activity against *S. marcescens* itself and therefore provide stronger pathogen-specific support than studies performed against unrelated bacteria. Nevertheless, murine and invertebrate models cannot reproduce the full pharmacokinetic, immunological, anatomical, and comorbidity-related complexity of human infection and cannot substitute for controlled clinical evaluation.
- Indirect translational lessons from other Gram-negative pathogens. Clinical and preclinical studies involving *Klebsiella pneumoniae*, *Pseudomonas aeruginosa*, *Acinetobacter baumannii*, and other Gram-negative pathogens should be interpreted separately as indirect translational evidence. Their principal relevance to *S. marcescens* lies in informing generalizable aspects of phage-therapy implementation—including isolate-specific phage matching, pulmonary delivery by nebulization, localized versus systemic administration, phage pharmacokinetics, cocktail design, combination with antibiotics, resistance monitoring, and safety assessment—rather than demonstrating pathogen-specific efficacy. For example, a 2026 comparative pilot study of nebulized phage therapy for MDR *K. pneumoniae* ventilator-associated pneumonia demonstrated that targeted phages could be incorporated into intensive-care workflows and delivered repeatedly through the respiratory route without identified treatment-related adverse events. Similarly, personalized nebulized phage therapy in patients with MDR *P. aeruginosa* pulmonary infection has provided clinical information concerning respiratory phage delivery, bacterial-burden monitoring, and phage–antibiotic treatment strategies. These observations strengthen the translational rationale for analogous approaches in *S. marcescens* pulmonary disease but cannot be extrapolated as evidence that comparable efficacy will occur in *S. marcescens*, because receptor usage, intracellular anti-phage defenses, bacterial physiology, biofilm architecture, phage replication kinetics, and antibiotic–phage interactions are pathogen- and strain-dependent.

The most compelling clinical evidence supporting phage therapy against MDR *Serratia marcescens* stems from a rigorously documented 2025 case report of a 59-year-old male with persistent postoperative pneumonia and empyema following lung cancer resection (Ronholm et al., 2016). After 120 days of unsuccessful antibiotic treatment with clindamycin, cefuroxime, levofloxacin, and amikacin/tazobactam, the patient remained culture-positive for an XDR *S. marcescens* strain in pleural fluid. Personalized therapy was initiated using the strictly lytic phage Spe5P4 at a concentration of 10^9^ PFU/mL, delivered via both intrapleural injection and nebulization, alongside meropenem and amikacin. Clinical improvement was rapid and sustained. Within 72 h, dyspnea and chest pain resolved, sputum production diminished, and oxygen saturation normalized. Serial chest computed tomography revealed progressive re-expansion of the left lung and near-complete resolution of pleural effusion by day 140. Quantitative microbial monitoring through 16S rRNA sequencing of pleural fluid showed a steady decline in *S. marcescens* abundance throughout phage administration. Importantly, no phage-resistant mutants were detected, and post-treatment isolates exhibited improved susceptibility to fluoroquinolones, with levofloxacin and ciprofloxacin MICs shifting from resistant to susceptible ranges. Safety evaluations confirmed the absence of hepatotoxicity, nephrotoxicity, or exacerbation of systemic inflammation. Inflammatory markers, including C-reactive protein and interleukin-6, decreased significantly after treatment initiation. No adverse events related to phage administration were observed during the 140-day follow-up. This case represents the first reported clinical application identified in this review of phage therapy specifically directed against MDR *Serratia marcescens*, incorporating pathogen identification, isolate-specific phage matching, multimodal phage administration, and clinical and microbiological monitoring. However, because phage Spe5P4 was administered concomitantly with meropenem and amikacin, the clinical and microbiological improvements cannot be attributed exclusively to phage therapy. The antibiotics may have contributed directly to bacterial clearance and may also have acted synergistically with the phage. Although the patient had not responded to prolonged antibiotic treatment before phage administration, the temporal association between initiation of the combined regimen and subsequent clinical improvement cannot establish an independent therapeutic effect of the phage. Accordingly, this report should be interpreted as an exploratory patient-level application demonstrating that individualized phage administration was clinically implementable in this case, rather than as evidence establishing the efficacy or generalizable clinical feasibility of phage therapy against *S. marcescens*. At present, this report appears to represent the only published patient-level evidence for phage therapy specifically directed against *S. marcescens*. It should therefore be described as an exploratory single-patient clinical application rather than a successful clinical trial. Although the temporal association between initiation of phage treatment and clinical improvement is encouraging, the absence of a control group and the concomitant administration of meropenem, amikacin, local drainage, and supportive care prevent the independent therapeutic contribution of phage Spe5P4 from being determined. Direct *S. marcescens*-specific preclinical studies provide additional proof-of-concept but remain limited. In a BALB/c mouse model of lethal *S. marcescens* infection, treatment with phage φSM9-3Y provided complete protection when administered immediately after bacterial challenge, whereas delayed treatment produced lower survival, demonstrating the importance of treatment timing and phage exposure. More recently, the lytic phage Sm10b_1 significantly improved survival in a *Galleria mellonella* model infected with a clinical bloodstream isolate of *S. marcescens*. These findings support *in vivo* activity against *Serratia*, but insect and murine infection models cannot substitute for controlled human studies. Taken together, the available *S. marcescens*-specific evidence remains limited and primarily supports biological plausibility and preclinical proof of concept. Direct human evidence is restricted to a single published patient-level therapeutic application, which demonstrates that phage administration was possible in that individual clinical setting but does not establish therapeutic efficacy or generalizable clinical feasibility. Organism-specific preclinical evidence is derived from only a small number of experimental infection models. Therefore, the present evidence base is insufficient to establish therapeutic efficacy, optimal dosing, preferred route of administration, treatment duration, or patient-selection criteria specifically for *S. marcescens* infection. Several important knowledge gaps remain. First, the host-range coverage of available *S. marcescens* phages has not been defined across sufficiently large and epidemiologically diverse collections of clinical isolates, particularly carbapenem-resistant and biofilm-forming strains. Second, the frequency, mechanisms, and clinical consequences of phage resistance during treatment remain poorly characterized. Third, *S. marcescens*-specific pharmacokinetic and pharmacodynamic data are lacking, including the relationships among administered phage dose, local phage concentration, bacterial burden, *in situ* amplification, treatment timing, and bacterial clearance. Fourth, direct evidence regarding phage–antibiotic interactions in *S. marcescens* remains limited, and synergy or antagonism should therefore be determined experimentally for individual phage–antibiotic combinations rather than inferred from other bacterial species. Finally, the activity of therapeutic phages against mature *S. marcescens* biofilms and under clinically relevant physiological conditions requires further investigation. Future studies should therefore concentrate on strengthening the *S. marcescens*-specific evidence base. Candidate phages should be evaluated against genetically, geographically, and clinically diverse isolate collections using standardized adsorption, efficiency-of-plating, and quantitative killing assays. Promising candidates should then be tested in reproducible infection models that reflect clinically important *S. marcescens* syndromes, including pneumonia, bloodstream infection, urinary tract infection, empyema, and device-associated infection. These studies should incorporate dose-ranging and treatment-timing experiments, serial measurement of viable phage and bacterial concentrations, assessment of resistance emergence, evaluation of phage–antibiotic interactions, and standardized safety endpoints. Such organism-specific data are required before controlled clinical studies can meaningfully establish the efficacy and optimal use of phage therapy against *S. marcescens.* Such studies may inform delivery routes, cocktail formulation, phage–antibiotic combinations, and clinical monitoring, but differences in bacterial receptors, anti-phage defense systems, biofilm architecture, and infection biology limit straightforward extrapolation between species.

A recent pilot study (Li et al., 2019) evaluated nebulized phage cocktails in intensive care patients with ventilator-associated pneumonia caused by carbapenem-resistant *Klebsiella pneumoniae*. The study demonstrated that bedside-prepared, GMP-compliant phage formulations could be safely administered via standard ventilator circuits, resulting in reduced bacterial burden in endotracheal aspirates and improved oxygenation indices without significant adverse effects. Although the target pathogen differed, the protocol’s emphasis on rapid phage matching, compatibility with critical care infrastructure, and localized pulmonary delivery provides a scalable framework directly applicable to *S. marcescens*-associated pneumonia. Further reinforcing this approach, a compassionate-use study of nine patients with cystic fibrosis infected with MDR *Pseudomonas aeruginosa* showed that nebulized phage therapy led to a median reduction of 10^4^ CFU/mL in sputum bacterial density between days 5 and 18 (Kohm et al., 2023). Preclinical models also support combination strategies. In a murine thigh infection model, high-dose AttikonH10 monotherapy (10^9^ PFU/animal) reduced bacterial burden by 1.09 log₁₀ CFU/thigh—comparable to amikacin (1.19 log₁₀)—demonstrating the potential efficacy of phage monotherapy against challenging Gram-negative infections (Tian et al., 2019). However, owing to the rapid reciprocal adaptation between phages and bacteria that fosters resistance development, phage monotherapy entails a substantial risk of therapeutic escape. As such, integrating phages with antibiotics has emerged as a foundational approach in modern phage therapy. The synergistic mechanisms underlying such combinations, including enhanced biofilm disruption, are considered to have broad applicability across bacterial species. Studies have shown that combining phages with antibiotics synergistically enhances the disruption of biofilms formed by *Escherichia coli* and *Pseudomonas aeruginosa*, leading to significantly greater reductions in both biofilm biomass and viable bacterial counts compared to either treatment alone (Vieira et al., 2021). In another study employing a hollow fiber infection model to mimic human pharmacokinetics, phage pretreatment followed by ciprofloxacin achieved >1,000-fold bacterial reduction and complete clearance without detectable resistance, unlike monotherapy. This highlights the potential of sequential phage–antibiotic regimens to suppress resistance evolution (Duan et al., 2025) (Table 1). Importantly, phage–antibiotic combinations are not universally synergistic; additive, neutral, and antagonistic interactions have all been reported, and the outcome is highly dependent on the specific phage–host–antibiotic combination, antibiotic concentration, multiplicity of infection, and sequence and timing of administration. Because productive phage infection depends on metabolically active bacterial cells and frequently exploits host transcriptional, translational, and DNA-replication machinery, antibiotics that strongly inhibit these cellular processes may impair phage replication. For example, inhibition of bacterial RNA polymerase by rifampicin can suppress replication of phages that depend on host transcriptional machinery, whereas protein-synthesis inhibitors, including some aminoglycosides, have been shown to inhibit early stages of phage infection and reduce phage amplification. In other phage–host systems, inhibition of DNA gyrase or topoisomerase activity has similarly reduced phage replication. Such antagonism can decrease progeny production, burst size, or overall bacterial killing and may therefore counteract the anticipated benefit of combination therapy. Consequently, phage–antibiotic synergy should not be assumed solely on the basis of the independent susceptibility of a bacterial isolate to both agents. Each clinically relevant phage–antibiotic pair should instead be evaluated experimentally across clinically achievable antibiotic concentrations and different treatment sequences, including simultaneous administration, phage-first exposure, and antibiotic-first exposure. These analyses should quantify both bacterial killing and phage replication, because an apparent reduction in bacterial density may conceal inhibition of phage amplification. Parameters such as viable bacterial counts, time–kill kinetics, phage titers, burst size, resistance emergence, and appropriate interaction models should therefore be considered when distinguishing synergy from additivity or antagonism. Where antagonism is observed during simultaneous treatment, sequential administration may provide an alternative strategy by allowing phage replication before bacterial metabolism is strongly suppressed by the antibiotic. For *S. marcescens*, available studies demonstrate that synergy can occur with selected phage–antibiotic combinations; however, direct systematic evidence defining antagonistic combinations in this species remains limited. Accordingly, antagonistic interactions demonstrated in other Gram-negative bacteria should be regarded as mechanistic and translational warnings rather than direct evidence that a particular antibiotic will antagonize an anti-*S. marcescens* phage. This uncertainty reinforces the need for isolate-, phage-, and antibiotic-specific combination testing before therapeutic administration. Taken together, the available *S. marcescens*-specific evidence supports biological plausibility and demonstrates preclinical *in vivo* activity, whereas direct human evidence remains restricted to a single published patient-level therapeutic application and is insufficient to establish clinical efficacy or generalizable clinical feasibility. Broader phage-therapy literature involving other Gram-negative pathogens provides indirect translational evidence suggesting that isolate-specific phage matching, localized administration, and selected phage–antibiotic combinations may influence therapeutic performance. However, these observations should not be interpreted as evidence that these approaches have established efficacy against *S. marcescens*, for which pathogen-specific clinical validation remains lacking. However, heterogeneity in phage preparations, dosing schedules, routes of administration, outcome measures, and concomitant antibiotic treatment prevents direct comparison among studies and precludes definitive conclusions regarding efficacy. Therefore, current evidence should be interpreted as hypothesis-generating rather than confirmatory. Prospective studies using standardized microbiological, pharmacokinetic, safety, and clinical endpoints are required to determine the independent therapeutic contribution of phages and to identify the patient populations most likely to benefit.

**Table 1:** Current *Serratia marcescens*-specific evidence for bacteriophage therapy and preclinical development.

Evidence level	Phage / study	Model and assessment	Key finding	Main limitation	Reference
Human clinical — exploratory single case	Spe5P4	One patient with refractory MDR *S. marcescens* pulmonary infection/empyema; intrapleural + nebulized phage with meropenem/amikacin	Clinical and microbiological improvement; no reported phage-related adverse events	Single uncontrolled case; independent phage effect cannot be determined	62
Preclinical *in vivo* — mammalian	φSM9-3Y	BALB/c mouse lethal infection model; phage administered at different post-infection times	Immediate treatment: 100% survival; 60-min delay: ~60% survival	Single murine model; no clinical validation	77
Preclinical *in vivo* — invertebrate	Sm10b_1 / Sm12	Clinical/environmental *Serratia* isolates; *Galleria mellonella* infected with UMH9	Sm10b_1 increased 6-day survival from 0% to 67%; Sm12 showed strain-dependent host range	Invertebrate model; Sm12 not therapeutically evaluated *in vivo*	78
Preclinical *in vitro* — phage–antibiotic interaction	vB_SmaP-SALSA	20 non-duplicate clinical isolates; host-range and liquid-killing assays	Productive lysis in 5/20 isolates; selected phage–antibiotic combinations prevented/overcame regrowth under tested conditions	Narrow host range; planktonic *in vitro* model	7
Preclinical *in vitro* — biofilm-related activity	UFV01	*S. marcescens* host; Enterobacteriaceae biofilm assays	Highly *S. marcescens*-specific; reduced biofilm formation in several *E. coli* strains without productive infection	*In vitro* evidence; therapeutic efficacy not demonstrated *in vivo*	61
*In vitro* — host-range/genomic characterization	PS2 (vB_SmaM_PS2)	Clinical *S. marcescens* isolates; host testing and whole-genome characterization	Lytic T4-like phage; 167,276-bp dsDNA genome	Characterization-focused study; no *in vivo* efficacy data	79
*In vitro* — host-range/genomic characterization	WSPA	MDR clinical *S. marcescens* isolates; host-range, growth, stability, genomic/proteomic analyses	Lytic activity against multiple MDR isolates; no identified virulence or AMR genes	Limited isolate panel; no *in vivo* or clinical validation	80

AMR, antimicrobial resistance; MDR, multidrug-resistant.

## 6 Discussion

The current evidence for phage therapy against multidrug-resistant *Serratia marcescens* should be interpreted according to a clear hierarchy. At the organism-specific level, it is established that multiple lytic phages can infect selected *S. marcescens* strains *in vitro*, that receptor usage and anti-phage defenses vary substantially among isolates, and that a limited number of phages have demonstrated antibacterial activity in experimental infection models. Direct human evidence, however, remains restricted to a single identified patient-level therapeutic application and therefore does not establish reproducible efficacy, optimal dosing, preferred routes of administration, or generalizable clinical feasibility. Accordingly, the principal translational question is no longer whether anti-*S. marcescens* phages can exhibit antibacterial activity, but whether that activity can be predicted, rapidly matched to individual clinical isolates, delivered effectively to the infection site, maintained despite bacterial and host-mediated resistance mechanisms, and reproduced under controlled clinical conditions. Consequently, the current *S. marcescens*-specific literature supports biological plausibility, limited preclinical *in vivo* activity, and demonstration that therapeutic administration is possible in an individual patient, while it does not establish reproducible clinical efficacy or generalizable clinical feasibility. The organism-specific evidence therefore remains at an early translational stage and consists predominantly of *in vitro* phage characterization, a limited number of experimental infection models, and a single identified patient-level therapeutic application. Clinical and preclinical findings reported for other Gram-negative pathogens should be considered separately as indirect translational evidence and should not be extrapolated as demonstrating therapeutic efficacy against *S. marcescens*. Consequently, the major challenge is to determine whether the promising experimental activity of anti-*S. marcescens* phages can be translated into reproducible, appropriately dosed, resistance-resilient, and clinically effective treatment.

The narrow and strain-dependent host range of many anti-*S. marcescens* phages makes rapid isolate-specific matching a central translational requirement rather than merely a logistical limitation. The single identified clinical application used individualized phage matching, but one case cannot establish the reliability of this strategy across diverse *S. marcescens* lineages. Conventional plaque, spot, and liquid-culture susceptibility assays remain the principal basis for generating phagograms; however, differences in assay conditions and interpretation currently limit interlaboratory reproducibility. Consequently, rapid and standardized phage-susceptibility testing capable of distinguishing productive infection from nonspecific bacterial clearing will be required if personalized phage selection is to become clinically actionable. Recent advances in phage therapy suggest that phenotypic phagograms may increasingly be complemented by bacterial and phage genomic information. For *S. marcescens*, potentially informative features include receptor-associated loci, lipopolysaccharide and capsular biosynthesis genes, CRISPR–Cas and other anti-phage defense systems, and genomic determinants associated with phage replication or resistance. Computational and artificial-intelligence-assisted approaches are also being explored in the broader phage field to predict phage–host compatibility and prioritize candidate phages for experimental testing. These approaches may reduce the search space and accelerate matching, but they should presently be regarded as complementary to, rather than replacements for, experimental susceptibility testing because clinically validated predictive models for *S. marcescens* have not yet been established. Establishing broader applicability will require systematic host-range studies, standardized susceptibility testing, and curated phage libraries representing the genetic and phenotypic diversity of contemporary clinical *S. marcescens* isolates. In settings lacking these resources, timely acquisition of effective phage preparations remains impractical. Developing standardized phage cocktails that cover dominant circulating clones—and integrating bacterial genomic features such as outer membrane receptor genotypes and defense system profiles into predictive susceptibility models—may improve accessibility (Paranos et al., 2025). Notably, certain *S. marcescens* phages encode depolymerases targeting capsular polysaccharides or lipopolysaccharides; these enzymes not only enhance penetration into biofilms but can also unmask cryptic phage receptors, thereby functionally broadening host recognition (Aframian and Eldar, 2023). This mechanism likely contributed to the rapid decline in bacterial load observed in intrapleural phage administration for empyema.

A major unresolved challenge is whether naturally occurring anti-*S. marcescens* phages can retain activity despite receptor heterogeneity, intracellular defense systems, and rapid bacterial adaptation. These barriers suggest that future phage development should move beyond selection based solely on plaque formation or short-term *in vitro* killing. Rational cocktails composed of phages with complementary host ranges, distinct receptor dependencies, and nonredundant susceptibility to bacterial defense systems may reduce the probability that a single resistance mechanism will abolish activity across the entire formulation. Phage engineering provides a complementary strategy. Advances in synthetic biology and genome editing now permit modification of phage host-recognition determinants, removal of undesirable genetic elements, introduction or optimization of depolymerase functions, and incorporation of genetic systems designed to enhance bacterial killing or circumvent specific resistance mechanisms. Engineered phages have entered clinical development for pathogens other than *S. marcescens*, demonstrating the broader translational feasibility of this approach. However, no comparable evidence currently establishes the safety or superiority of engineered anti-*S. marcescens* phages. Their potential application therefore requires organism-specific validation of genetic stability, host range, resistance evolution, off-target effects, manufacturing consistency, and biosafety.

Moreover, phage pharmacokinetics (PK) and pharmacodynamics (PD) differ fundamentally from those of conventional antibiotics because therapeutic phages are self-replicating biological agents whose concentrations may change dynamically in the presence of susceptible bacteria. From a PK perspective, therapeutic exposure is determined by the administered dose and route, distribution to the infected compartment, tissue penetration, adsorption to susceptible bacteria, *in situ* replication, and elimination or inactivation by host factors. Following systemic administration, circulating phage concentrations may decline because of tissue distribution, sequestration by the mononuclear phagocyte system, particularly in the liver and spleen, and immune-mediated neutralization. In contrast, localized administration, such as intrapleural or inhaled delivery, may generate higher phage concentrations at anatomically confined infection sites while reducing losses associated with systemic distribution and clearance. Consequently, plasma phage concentrations alone may not accurately represent exposure at the site of infection. Phage PK is additionally target-dependent and potentially nonlinear. When a sufficient density of metabolically active and susceptible bacteria is present, productive infection may generate progeny phages and increase local phage concentrations through *in situ* amplification. Conversely, when bacterial density is low, receptors are inaccessible, bacterial physiology is unfavorable for infection, or host clearance is rapid, phage replication may be insufficient to compensate for phage loss. Thus, the administered PFU dose does not necessarily predict the concentration or persistence of active phage at the infection site. Adsorption rate, latent period, burst size, bacterial density, phage stability, and emergence of resistant bacterial subpopulations all influence this dynamic exposure profile. From a PD perspective, therapeutic activity should be assessed by relating phage exposure to quantitative changes in the target bacterial population. Relevant endpoints include the magnitude and rate of CFU reduction, duration of bacterial-growth suppression, time to bacterial regrowth, changes in viable phage titer, production of progeny phages, and emergence of phage resistance. Multiplicity of infection (MOI) describes the initial phage-to-bacterium ratio in experimental systems but should not be regarded as the sole PK/PD determinant because the effective number of productive infections depends on adsorption efficiency, bacterial physiological state, spatial accessibility, and phage clearance. Therefore, absolute PFU and CFU concentrations, timing of phage administration, and serial changes in both bacterial and phage populations should be reported alongside MOI. Unlike antibiotics, for which indices, such as Cmax/MIC, AUC/MIC, and time above the MIC are routinely used, no universally validated PK/PD index or susceptibility breakpoint currently predicts therapeutic phage efficacy. Accordingly, rational phage dosing should ideally integrate serial measurements of viable phage concentrations and bacterial burden at clinically relevant sites rather than relying only on the administered phage dose. For *S. marcescens*, pathogen-specific PK/PD data remain particularly limited, and the available Spe5P4 clinical application does not establish validated exposure targets, optimal dosing intervals, or generalizable dose–response relationships. Future studies should therefore prospectively characterize phage concentration–time profiles, bacterial clearance kinetics, local phage amplification, resistance emergence, and their relationship with clinical outcomes. A further critical limitation involves the dual risk of bacterial resistance development and neutralization of therapeutic phages by the host immune system. Although phages possess the capacity to co-evolve with their bacterial hosts, the timescale required for such adaptive responses typically exceeds the narrow therapeutic window in acute clinical settings. Of additional concern is the potential development of neutralizing anti-phage antibodies following repeated phage administration. Preclinical animal studies have demonstrated that repeated exposure to therapeutic phages can induce neutralizing antibody responses capable of reducing phage activity and persistence. However, the clinical significance of such immune neutralization in humans remains incompletely defined and should not be inferred directly from animal data. Localized delivery routes, such as nebulization or intrapleural administration, may theoretically reduce systemic phage exposure and thereby limit systemic immune recognition; however, the extent to which route of administration modifies anti-phage immunogenicity in humans remains insufficiently characterized. In addition, phage-derived nucleic acids may interact with innate immune receptors, including Toll-like receptor 9 (TLR9), providing a plausible mechanism for inflammatory immune activation; however, the clinical relevance and magnitude of such responses during therapeutic phage administration remain uncertain. This aspect warrants rigorous evaluation in future controlled trials to ensure both safety and sustained therapeutic activity.

Current evidence derives largely from uncontrolled compassionate-use reports, making it difficult to disentangle the specific contribution of phage therapy from concomitant antibiotics or supportive care. This limitation is particularly relevant because most reported patients received phages in combination with one or more antibiotics. Consequently, the observed therapeutic response may reflect independent antibiotic activity, independent phage activity, or an additive or synergistic interaction between the two. Future clinical studies should therefore include antibiotic-only comparator groups, standardized combination regimens, and serial measurements of bacterial burden, phage concentrations, and antibiotic exposure to distinguish the contribution of each treatment component. Future studies should employ prospective, controlled trial designs to define the independent efficacy and safety of phage-based interventions in well-defined indications—such as carbapenem-resistant *S. marcescens* empyema or catheter-related bloodstream infections. Concurrently, longitudinal assessments of the impact on respiratory or gut microbiota are needed to identify risks of secondary ecological disruption.

Clinical translation is also constrained by manufacturing and regulatory challenges. Each therapeutic phage should undergo comprehensive genomic characterization to confirm identity, genetic stability, strictly lytic activity, and the absence of virulence, antimicrobial-resistance, or other undesirable genes. Manufacturing processes should additionally establish formulation and storage conditions that preserve phage viability and potency throughout the intended shelf life. For personalized phage preparations, standardized production procedures and predefined release criteria are needed to ensure reproducibility in identity, titer, purity, sterility, and endotoxin content across batches. Phage cocktails require additional quality control to verify the identity and relative abundance of each component, overall potency, stability, and absence of unintended phage–phage interactions. Regulatory oversight is particularly challenging because personalized therapy may require rapid modification of cocktail composition in response to changes in bacterial susceptibility or phage resistance; therefore, adaptive regulatory frameworks should permit clinically justified composition changes while maintaining consistent requirements for genomic safety, manufacturing quality, and batch release. Therapeutic phages are biologically dynamic agents whose host range, genomic composition, and susceptibility profiles may remarkably differ among preparations. Regulatory frameworks must therefore accommodate the rapid modification of personalized phage cocktails while maintaining rigorous requirements for genomic safety, identity, purity, potency, endotoxin removal, sterility, and batch-to-batch consistency. The establishment of harmonized quality-control standards, accredited phage banks, validated susceptibility-testing methods, and expedited pathways for updating cocktail composition will be essential for integrating precision phage therapy into routine clinical practice.

Despite encouraging mechanistic findings and limited organism-specific preclinical evidence, the current evidence base is insufficient to establish the clinical efficacy of phage therapy against multidrug-resistant *S. marcescens*. Most *S. marcescens*-specific evidence remains preclinical, consisting of phage isolation and characterization studies, *in vitro* susceptibility experiments, biofilm-related investigations, and a small number of animal or invertebrate infection models. Human evidence is presently represented by a single identified patient-level therapeutic application. Accordingly, neither response rates, reproducibility of clinical benefit, optimal dosing, comparative efficacy, nor treatment-associated risks can be estimated reliably from the existing literature. The available evidence therefore supports biological plausibility and provides an initial demonstration that clinical administration is possible, but it does not establish clinical efficacy or generalizable clinical feasibility.

Several important knowledge gaps must be addressed to advance clinical translation. These include incomplete identification of clinically relevant phage receptors, limited understanding of bacterial defense systems and resistance evolution *in vivo*, insufficient pharmacokinetic and pharmacodynamic data, uncertain effects of host immunity and neutralizing antibodies, and the absence of standardized methods for phage susceptibility testing and cocktail selection. Future research should prioritize multicenter prospective studies with harmonized microbiological and clinical endpoints, antibiotic-only and combination-treatment comparator groups, longitudinal monitoring of phage and bacterial populations, and systematic assessment of safety and microbiome effects. Precision phage therapy based on rapid genomic characterization of the infecting isolate, curated phage libraries, receptor-informed cocktail design, and engineered phages carrying optimized depolymerases or anti-defense functions may further improve host coverage and therapeutic reliability. Parallel development of scalable manufacturing procedures and adaptive regulatory pathways will also be essential for converting individualized rescue therapy into a reproducible clinical platform.

In summary, the available literature supports the biological plausibility of phage therapy against MDR *S. marcescens* and provides limited organism-specific preclinical evidence together with a single exploratory human application. However, the current evidence is insufficient to establish clinical feasibility or therapeutic efficacy. Its broader adoption, however, hinges on a deeper mechanistic understanding of pathogen–phage interactions—including receptor engagement, bacterial defense architectures, and phage-encoded countermeasures—combined with rational engineering of phages incorporating functional depolymerases or anti-defense effectors, the establishment of robust pharmacokinetic–pharmacodynamic frameworks, and validation through high-quality clinical trials that quantify net therapeutic benefit. Only through such an integrated effort can phage therapy evolve from an experimental salvage measure into a standardized component of antimicrobial care.

## 7 Conclusion

Multidrug-resistant *Serratia marcescens*, particularly carbapenem-resistant strains, remains a difficult-to-treat pathogen associated with limited therapeutic options and poor clinical outcomes. Phage therapy represents a biologically plausible and potentially useful therapeutic strategy because of its strain-specific bactericidal activity, capacity to target biofilm-associated bacteria, and potential interaction with antibiotics. However, direct clinical evidence for phage therapy against *S. marcescens* remains extremely limited and is currently based primarily on isolated compassionate-use experience and a small body of preclinical research. Therefore, its efficacy as either monotherapy or adjunctive treatment has not yet been established.

Clinical translation is further constrained by the narrow host range of several phages, variability among clinical isolates, emergence of phage resistance, bacterial anti-phage defense systems, uncertain pharmacokinetic and pharmacodynamic behavior, and possible immune-mediated phage neutralization. The absence of standardized susceptibility testing, manufacturing procedures, quality-control criteria, and regulatory pathways also limits reproducibility and broader clinical implementation.

Future research should prioritize the evaluation of well-characterized anti-*S. marcescens* phages and receptor-diverse cocktails in clinically relevant infection models, systematic assessment of phage–antibiotic combinations, and prospective controlled clinical studies with standardized microbiological and clinical endpoints. Genomically informed phage selection, curated phage libraries, and carefully engineered phages may improve host coverage and therapeutic consistency, but their safety and efficacy require rigorous validation. At present, phage therapy should be regarded as an investigational and potentially complementary approach rather than an established alternative to conventional antimicrobial treatment for MDR *S. marcescens* infections.
